# Primer Sets Developed for Functional Genes Reveal Shifts in Functionality of Fungal Community in Soils

**DOI:** 10.3389/fmicb.2016.01897

**Published:** 2016-11-29

**Authors:** S. Emilia Hannula, Johannes A. van Veen

**Affiliations:** ^1^Department of Microbial Ecology, Netherlands Institute of Ecology (NIOO-KNAW)Wageningen, Netherlands; ^2^Insititute of Biology, Leiden UniversityLeiden, Netherlands

**Keywords:** fungi, soil fungal community, functional genes, glycosyl hydrolases, soil microbiology, peroxidases, chronosequence

## Abstract

Phylogenetic diversity of soil microbes is a hot topic at the moment. However, the molecular tools for the assessment of functional diversity in the fungal community are less developed than tools based on genes encoding the ribosomal operon. Here 20 sets of primers targeting genes involved mainly in carbon cycling were designed and/or validated and the functioning of soil fungal communities along a chronosequence of land abandonment from agriculture was evaluated using them. We hypothesized that changes in fungal community structure during secondary succession would lead to difference in the types of genes present in soils and that these changes would be directional. We expected an increase in genes involved in degradation of recalcitrant organic matter in time since agriculture. Out of the investigated genes, the richness of the genes related to carbon cycling was significantly higher in fields abandoned for longer time. The composition of six of the genes analyzed revealed significant differences between fields abandoned for shorter and longer time. However, all genes revealed significant variance over the fields studied, and this could be related to other parameters than the time since agriculture such as pH, organic matter, and the amount of available nitrogen. Contrary to our initial hypothesis, the genes significantly different between fields were not related to the decomposition of more recalcitrant matter but rather involved in degradation of cellulose and hemicellulose.

## Introduction

Fungi are ubiquitous and diverse; the global number of fungal species has been estimated to be more than 1.5 million species (Hawksworth and Rossman, 1997; Hawksworth, 2001). At the local scale, fungal diversity is thought to have important consequences for ecosystem functioning particularly through their contribution to nutrient cycling and transport, moisture retention, and plant growth (van der Heijden et al., 1998; Setälä and McLean, 2004). Despite the presumptive great importance of the relationship between fungal diversity and ecosystem function (Nielsen et al., 2011), little is known about extent of the diversity even at the local scale and about the factors generating and maintaining it mainly because the majority of the species are not yet been described and are potentially unculturable. Next-generation amplicon sequencing technique targeting for example intergenic transcribed spacer (ITS)-region have greatly improved our understanding of the community diversity (Meiser et al., 2014; Tedersoo et al., 2014) but this technique offers little insight into function.

Traditionally, fungi have been divided into discrete ecological guilds, such as leaf litter-decomposers, humus saprobes, white-, and brown-rot wood decaying fungi, parasites, and mycorrhizal symbionts (Carlile et al., 2001). However, the functionality of individual species, and the synergistic effects among them are often obscure. For instance, from the well-studied wood-decaying systems it is known that fungal species differ markedly in the way they decompose wood (Worrall et al., 1997), with the decomposition rates depending on the fungal community structure (Boddy et al., 1989). Furthermore, every ecosystem has many fungal types with overlapping functions (Liers et al., 2011).

There is a clear succession in the communities that are involved in the decomposition of litter and organic matter. First, when fresh material is released to the system, r-strategists (both bacteria and fungi) will appear in high numbers, and after the easily degraded material is gone, K-strategists will start the more complicated degradation processes (de Boer et al., 2005). For these steps different sets of genes are required to produce enzymes needed to breakdown the organic material. The first colonizers include yeasts, zygomycetes, and several ascomycetes that produce cellulases and use easily available soluble compounds before being replaced by asco- and basidiomycetes that produce ligninolytic enzymes and glycosyl hydrolases (GHs) to attack the insoluble and more recalcitrant substrates such as lignin. Cellulases play a key role in organic carbon turnover in soil ecosystems (Rabinovich et al., 2002). The ability to degrade cellulose is distributed across the entire fungal kingdom (Lynd et al., 2002) while the ligninolytic enzymes (Wong, 2009) such as laccases, lignin peroxidases, and manganese peroxidases (Martinez et al., 2009; Edwards et al., 2011) are produced only by certain specialized saprotrophic fungi and especially basidiomycetes (Paul, 1989; Liers et al., 2011). Most of the actions are however synergistic: easily degradable cellulose from plant cell walls, an important source of energy for saprotrophic micro-organisms, is bundled into crystalline fibrils, which are linked by hemicellulose to form larger, lignin-encrusted microfibrils (Koshijima and Watanabe, 2003). Thus, a community of fungi and bacteria with different enzymatic capabilities rather than individual species is needed to break down all the cellulose, hemicellulose, and lignin.

Recent studies have confirmed that the content and composition of the gene families encoding enzymes involved in decomposition processes such as fungal oxidative lignin enzymes (FOLy) and carbohydrate-active enzymes (CAZy; Lombard et al., 2014) could be used as functional indicators for fungi (Eastwood et al., 2011; Floudas et al., 2012). Glycosyl hydrolases (EC 3.2.1-) are genes primary involved in cellulose and hemi-cellulose degradation. The division into GH families is based on functionality of the enzymes produced and different clades may be identified through sequences of the functional genes encoding the enzymes and subsequently their prevalence in the environment assessed. Furthermore, although bacteria and archaea are assumed to be the most important microbes participating in nitrogen cycling, fungi are known to contribute to the cycling of nitrogen as well. Fungal contribution to nitrogen cycling in this system was evaluated using nitrate reductase (NIAD) gene composition and fragment richness (Gorfer et al., 2011).

The sequencing of genomes of various species of fungi (http://1000.fungalgenomes.org/) has enabled us to investigate the presence and types of functional genes and subsequently design probes to target as many fungal groups as possible. Despite the increased capacity and decreased price of sequencing, due to the low number of transcripts of fungal genes involved in plant cell wall degradation, making up often <1% of the metatranscriptomic library (Damon et al., 2012), targeted approaches are still needed in ecological studies in order to compare statistically meaningful sample sets with each other. Therefore, the main objectives of this study were to characterize major fungal gene fragments associated with degradation of organic matter in grassland soils and to study if conversion of fields from agricultural used soils to species rich grasslands affects the diversity of selected genes.

To meet these objectives, we developed new primer sets and validated existing ones targeting functional genes and applied these to study the fungal functionality in chronosequence of abandoned agricultural land. The genes included in this study were selected based on existing metagenomics and metatranscriptomic information of soils and are thought to be the types most commonly found in soils (Kellner et al., 2010; Damon et al., 2012). This system has earlier been characterized to have a stable fungal biomass after initial increase after 2 years since abandonment from agriculture (Van der Wal et al., 2006). The fungal community structure has been shown to be different between the short term and long term abandoned fields in an earlier study (Thomson et al., 2015) but the functionality of fungi is not yet studied. The fields included in this study were abandoned for either <10 years or over 25 years. We hypothesized that the fields abandoned for longer time would have more genes coding for enzymes involved in the degradation of recalcitrant organic matter and the recently abandoned fields would have more fungi capable of producing enzymes degrading simple sugars. Furthermore, we hypothesize that the diversity and richness of genes would increase along the secondary succession gradient.

## Materials and methods

### Field sites and sampling

We selected six fields of which three were abandoned from agricultural use for <10 years [fields Reijerskamp (RE) and Oud Reemst (OR) for 6 years and field Telefoonweg (TW) for 10 years] and three sites for over 25 years ago [Mosselseveld (MV) for 26 years and Boersbos (BB) and Dennenkamp (DE) for 29 years, respectively]. All fields, located in the Veluwe region in the Netherlands, were managed by low-intensive grazing of natural and introduced herbivores (roe deer, fallow deer, red deer, horses, Scottish Highland cattle) to prevent forest development (Van der Wal et al., 2006). Details on the sampling locations, soils, and the soil properties are presented in Table S1.

Three replicate cores of 5 cm diameter and 20 cm deep were collected from the six fields and kept separate. The total number of samples was thus 18. The soil was sieved (4 mm mesh size) and stored at −20°C for molecular analyses. Fresh soil was used to culture fungi in order to obtain pure fungal cultures from each soil type.

### DNA extractions from soil and fungal cultures

DNA from soils was extracted from ~0.5 g of soil (wet weight) with a Power Soil DNA isolation kit (MOBIO Laboratories, Inc.) using a bead beating system. Yields of genomic DNA were inspected on 1% agarose gel and visualized under UV after ethidium bromide staining.

Pure fungal cultures were obtained from sieved fresh soil collected from the same soils by culturing them in three different media. The media used were water agar (WA), 1/10 potato dextrose agar (PDA), and basidiomycete specific media (Blanchette et al., 2010) with added TRITON-X100 (Sigma-Aldrich) to prevent colony expansion. Representative colonies were collected and grown on respective media without added TRITON-X100. The DNA from these fungi was extracted using Zymo Research Fungal/Bacterial DNA kit according to manufacturer's instructions with added β-mercaptoethanol.

### Primer design and validation

The target genes tested are presented in Table 1. These genes were selected based on their presence in soils and availability of primers. If primers were not found in literature but the gene was found to be common in grassland soils in previous metagenomics studies, an attempt to design new primers was made. In addition to the genes included in the study, two other genes (namely GH1 and GH61) were evaluated for possible primer sites, but as such were not found (due to similarity with bacterial sequences or lack of conservation) these genes were not investigated further. The primers designed in this study are based on 21–49 sequences (depending on the available sequences per gene) of desired gene from fungal origin in CaZY database in November 2012. CaZY database was selected as it is a curated database for carbohydrate genes. In order to reach the best possible coverage for the primers, sequences from all fungal orders represented in CaZY were used in analyses and bacterial and plant genes were used as outgroups. All sequences from desired gene origin were aligned using MEGA4 software and conserved regions were detected visually. Primers were designed to amplify coding regions by using known protein sequences as references. The primers were made as degenerate possible to cover most fungal taxa possible. Gradient PCRs were performed for DNA from pure cultures and soil DNA with the newly developed primers to determine annealing temperatures and if multiple options of primers were designed, best one was selected (Table S2). The criteria for selection of best primer combination were: amplification of as many fungal pure cultures as possible and a clear PCR product from the soil samples. All the primers found from literature were further tested and validated using eight pure cultures of fungi. The optimized PCR conditions and primer sequences are presented in Table S2 and detailed information on primer design in Supplementary File 1. All PCRs were performed with positive (DNA isolated from fungal pure culture) and negative controls.

**Table 1:** **Name used for the gene, enzyme it codes and reference for the primer set used**.

**Name**	**Enzyme target**	**References for primers**
GH3	β-D-glucosidases (3.2.1.21) or xylan 1,4-beta-xylosidase (3.2.1.37)	Kellner et al., 2010
GH5	β-mannosidase (3.2.1.25) is a cellulase	Kellner et al., 2010
GH6	Cellobiohydrolase II: endoglucanase (EC 3.2.1.4) and cellobiohydrolase (EC 3.2.1.91)	This study/Kellner et al., 2010
GH7	Cellobiohydrolase I: endoglucanase (EC 3.2.1.4) and cellobiohydrolase (EC 3.2.1.91)	Edwards et al., 2008
GH10	Endo-beta-1,4-xylanases (EC 3.2.1.8)	Kellner et al., 2010
GH11	Endo-beta-1,4-xylanases (EC 3.2.1.8)	Kellner et al., 2010
GH15	Glucoamylase (EC 3.2.1.3)	This study
GH18	Chitinase (EC 3.2.1.14)	This study
GH31	α-glucosidases (EC 3.2.1.20), simple sugar using	Kellner et al., 2010
GH45	Endoglucanases (EC 3.2.1.4)	Kellner et al., 2010
GH51	alpha-L-arabinofuranosidase (EC3.2.1.55)	Kellner et al., 2010
GH74	oligoxyloglucan reducing end-specific cellobiohydrolase (3.2.1.150)	Kellner et al., 2010
GH76	α-1,6-mannanase (EC 3.2.1.101)	This study
Mn-Peroxidase	Class II peroxidases, lignin peroxidases (EC 1.11.1.13) and Mn-peroxidases (EC 1.11.1.14)	Bödeker et al., 2009
Laccase	Benzenediol: oxygen oxidoreductase (EC 1.10.3.2	Kellner et al., 2009
Oxalate decarboxylase	Oxalate decarboxylase (4.1.1.2)	Kellner et al., 2010
Cellobiosedehydrogenase	Involved in degradation of cellulose (EC 1.1.99.18)	This study
Heme-thiolate peroxidase	Heme-thiolate peroxidase (EC1.11.2.-) or chloroperoxidase (EC1.11.1.10)	Kellner et al., 2010
Glucose oxidase	Glucose oxidase (EC 1.1.3.4)	This study
Nitrate reductase	Enzyme involved in nitrate uptake by higher fungi (EC 1.7.99.4)	Gorfer et al., 2011

PCR products from all 18 soil samples (6 soils and three replicates from each soil) were purified using Qiaqen PCR purification kit and cloned into *Escherichia coli* JM109 using the pGem-T Easy System II cloning kit (Promega, UK) with a vector: insert ratio of 3:1. Approximately 40 successful transformants per sample were selected for amplification and further 24 clones per soil were sequenced. The selection of clones to be sequenced was based on covering as many types as possible by selecting amplicons of different lengths. If multiple fragments of same size were present, the clones were selected randomly. In total, 144 clones per functional gene were sequenced per sample resulting to total of around 2500 clones sequenced.

### Sequence analyses

Nucleotide sequences in a same sample showing 97% similarity to each other were classified into operational taxonomic units (OTUs) by aligning the sequences with each other (with ClustalW) and calculating their pairwise similarity in MEGA4 (Tamura et al., 2007). This was done to calculate the richness of the unique OTUs per sample. OTUs covering >10% of the library per treatment were defined as dominant. Minimum of eight reference sequences from each gene family were retrieved from CAZY database and trimmed in MEGA4 to correspond the length of sequences obtained in this study (reference strains presented in Table S3). The sequences were aligned with each other using ClustalW (Larkin et al., 2007) to determine their similarities across samples and to the reference sequences. Taxonomic assignment of sequences was performed 75, 85, 90, and 95% for order, class, family and genus designations, respectively (Chen et al., 2013). Unique sequences (aligned across samples in order to determine one representative sequence for each type) were deposited in NCBI GenBank under accession numbers -KP749176.

In order to evaluate the composition of protein sequences, Augustus software was used to predict the partial protein (and mRNA) sequences from the DNA sequences using fungal genomes as a reference to enable correct annotation of the coding region (Keller et al., 2011). The sequences that did not code a protein when compared with multiple fungal strains were discarded from the mRNA data-set. The similarity of the obtained proteins was compared to known protein sequences obtained from the CaZy database in a similar way than was done for nucleotide sequences.

### Statistical analyses

UniFrac-weighted PCA (weighted using number of OTUs in a sample) was applied to examine differences in the functional communities between soil samples (Lozupone and Knight, 2005) using the data on unique OTUs in a sample and the information on how many times this sequence was present in the sample. Trees were built using ClustalW aligned data and exported to UniFrac.

The dissimilarities in gene composition between samples were calculated based on their UniFrac distance and correlated with each other and with dissimilarities between samples in soil physio-chemical factors measured, using linear correlation. Dissimilarities in plant communities which was a multivariate data-set were calculated using Principle Component Analyses (PCA) and calculating the dissimilarities between treatment for the two main axis. The two first axis explaining together 79% of the total variation in plant cover were used to calculate the dissimilarities in plant community between samples. The differences in richness of the genes and genes assigned into function were evaluated using the one-way analyses of variance testing. The assumption of normality was tested with Shapiro–Wilk statistics and homogeneity of variances was assessed with Levene's test. Differences between short-term and long-term abandoned fields were tested with Tukey's HSD test.

## Results

The primers in this study were designed to catch as broad spectrum of fungi as possible (see Supplementary File 1 for details). For example, the cellobiose dehydrogenase primers amplified *in silico* 25 of the 29 selected reference strains from 15 (14) orders. Only *Stachybotrys bisbyi* (Hypocreales), *Grifola frondosa* (Agaricales), and two Verticillium species (Glomerellales) had mismatches in their cellobiose dehydrogenase gene which could prevent primer attachment. *In vitro*, these primers amplified five out of the eight fungal strains tested. The selected glucose oxidase primers amplified *in silico* 19 out of the selected 21 strains and *in vitro* seven out of eight fungi. The details on the performance of all the primers designed can be found in the Supplementary File 1. All existing primers were tested with the same eight pure cultures of soil fungi for their specificity (Table S3). Some primers amplified all the selected cultured fungi while some were specific to groups or phyla. Details on the specificity can be found in Table S3.

After optimization of methods, the PCRs for the soils gave positive signal for all except one gene tested. Unfortunately we could not obtain enough PCR products from three of the soils (MV, TW, and BB) for successful cloning of the endoglucanase gene GH45 and this gene was excluded from further analyses. The specificity of the other primers varied and the percentage of sequences that could be correctly assigned to the desired gene family was between 62.5% for oxalate decarboxylase to 100% for seven gene fragment types (GH5, GH18, GH31, GH51, GH74, GH76, and glucose oxidase). The percentage of sequences predicted by Augustus to code a protein sequence was between 52.5% for GH5 and 88.3% for nitrate reductase genes. The sequences that did not code a (partial) protein were removed from the dataset and not included further in the analysis.

**Figure 1:**
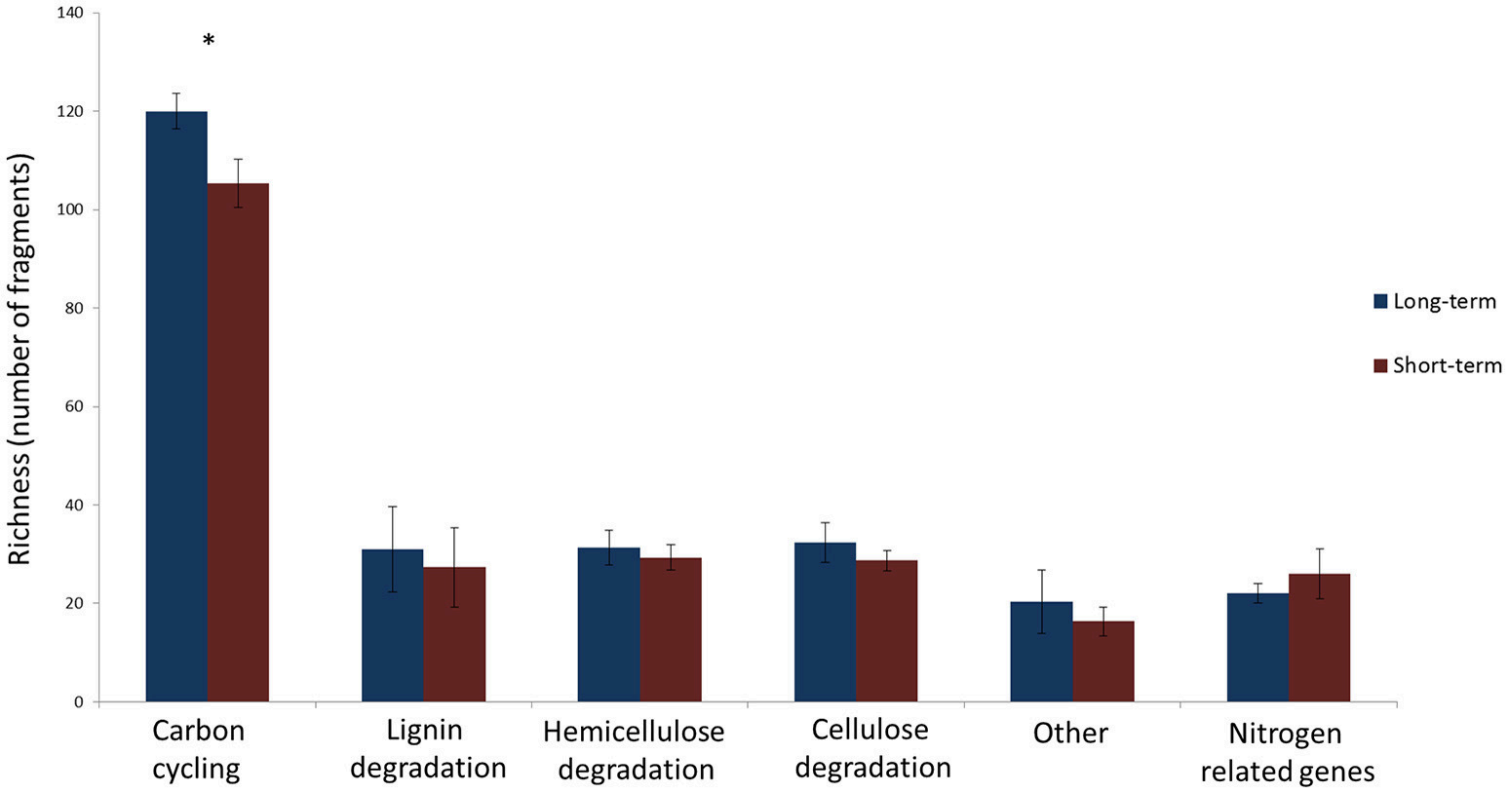
**The number of unique gene fragments determined as number of unique sequences in a sample in long-term (blue) and short-term (red) abandoned fields**. The bars represent the averages of 3 fields per treatment and error bars depict standard deviation. The list of how genes are divided into categories is presented in Table 2. Statistically significant differences (one-way ANOVA) between long-term and short-term abandoned fields are marked with asterisks.

The number of unique sequence types obtained per sample ranged from a single dominant type of GH51 fragment in field RE to 18 types of GH18 fragments in field DE, respectively (Table 2). There were differences in the number of unique types between soils but the richness of none of the genes studied was significantly affected by the time since abandonment (Table 2). However, when looking at groups of genes together clustered by function, we observed more genes involved in degradation of lignin, cellulose and hemicellulose in fields abandoned for longer than 25 years [Figure 1; *F*_(1, 4)_ = 9.3, *p* = 0.038].

**Table 2:** **The number of unique fragments of each gene studied detected in each field and averaged over treatments**.

		**GH3**	**GH5**	**GH6**	**GH7**	**GH10**	**GH11**	**GH15**	**GH18**	**GH31**	**GH51**	**GH74**	**GH76**	**Glucose oxidase**	**Cellobiose dehydrogenase**	**Hemethio peroxidase**	**Laccases**	**Oxalate decarboxylase**	**Mn-Peroxidases**	**NIAD 12R**	**NIAD 13R**
Long-term	BB	7	4	9	9	5	16	4	10	11	4	2	3	2	2	8	9	6	10	10	10
	MV	2	6	6	9	13	12	2	5	2	2	2	1	5	3	15	13	11	14	12	10
	DE	7	3	11	10	8	18	3	9	2	4	3	2	6	2	5	3	11	9	11	13
Short-term	RE	2	7	6	7	13	11	2	7	3	1	5	3	6	4	6	3	9	10	12	9
	OR	2	2	4	12	12	10	2	8	2	2	3	3	6	5	9	7	16	8	18	13
	TW	4	2	9	8	11	16	3	2	2	2	2	1	6	2	10	4	6	8	12	14
	Long-term	5.3	4.3	8.7	9.3	8.7	15.3	3.0	8.0	5.0	3.3	2.3	2.0	4.3	2.3	9.3	8.3	9.3	11.0	11.0	11.0
	Short-term	2.7	3.7	6.3	9.0	12.0	12.3	2.3	5.7	2.3	1. 7	3.3	2.3	6.0	3.7	8.3	4.7	10.3	8.7	14.0	12.0

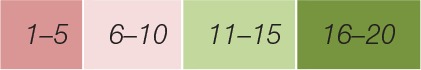

As measured by using the calculated UniFrac significant differences, the short-term abandoned fields differed significantly from the long-term abandoned fields in the composition of GH3, GH6, GH10, GH31, heme-thiolate peroxidases, and nitrate reductases (Table 3). In most cases species composition in an individual fields was unique to the field and phylogenetically similar gene-types were not shared within abandonment category (Table 3, **Figure 3**). The relationship of the samples from the short term and long-term abandoned fields is reflected in the PCA plots (**Figure 3**).

**Table 3:** **UniFrac significances between treatments based on both nucleotide and protein distances**.

	**Nucleic acid based**	**Protein based**	**Function**
	**Differences between long-term and short-term abandoned fields**	**Differences between long-term and short-term abandoned fields**	
**CARBON RELATED GENES**			
**Lignin related**			
Mn-Peroxidase	*p* = 0.06	*p* = 0.12	Mn-Peroxidase
Laccases	*p* = 0.8	*p* = 0.87	Laccase
Oxalate decarboxylase	*p* = 0.24	*p* = 0.60	Oxalate decarboxylase
Cellobiosedehydrogenase	*p* = 0.10	*p* = 0.79	Cellobiosedehydrogenase^*^
**ENXYME OXIDIZING AROMATICS**			
Heme-thiolate peroxidase	***p* = 0.04**	***p* = 0.03**	Heme-thiolate peroxidase
**HEMICELLULOSE/CELLULOSE RELATED**			
**Cellulose related**			
GH3	***p* = 0.000**	*p* = 0.10	Cellulase or xylanase
GH5	*p* = 0.14	*p* = 0.21	Cellulase
GH6	***p* = 0.000**	***p* = 0.03**	Cellobiohydrolase II
GH7	*p* = 0.59	*p* = 0.31	Cellobiohydrolase I
GH74	*p* = 0.18	n.d.	Oligoxyloglucan
Cellobiosedehydrogenase	*p* = 0.10	*p* = 0.79	Cellobiosedehydrogenase^*^
**Hemicellulose related**			
GH3	***p* = 0.000**	*p* = 0.10	Cellulase or xylanase
GH10	***p* = 0.000**	***p* = 0.01**	Xylanase
GH11	*p* = 0.12	*p* = 0.16	Xylanase
GH51	*p* = 0.66	*p* = 0.27	Xylan sidechain degradation
GH76	*p* = 0.06	*p* = 0.10	Xylanase
**Other**			
GH15	*p* = 0.07	*p* = 0.10	Glucoamylase, starch metabolism
GH18	*p* = 0.07	*p* = 0.06	Chitinase
GH31	***p* = 0.01**	***p* = 0.04**	α-glucosidases, starch metabolism
Glucose oxidase	*p* = 0.32	*p* = 0.33	Breakdown of mannose, glucose and xylose
**NITROGEN RELATED GENES**			
Niad12R &13R	***p* = 0.000**	*p* = 0.08	Fungal nitrate uptake

Significant values are marked with bold.

* Cellobiosedehydrogenases are considered both as lignin related and cellulose related genes as the reaction creates an active hydroxyl radical.

**Figure 2:**
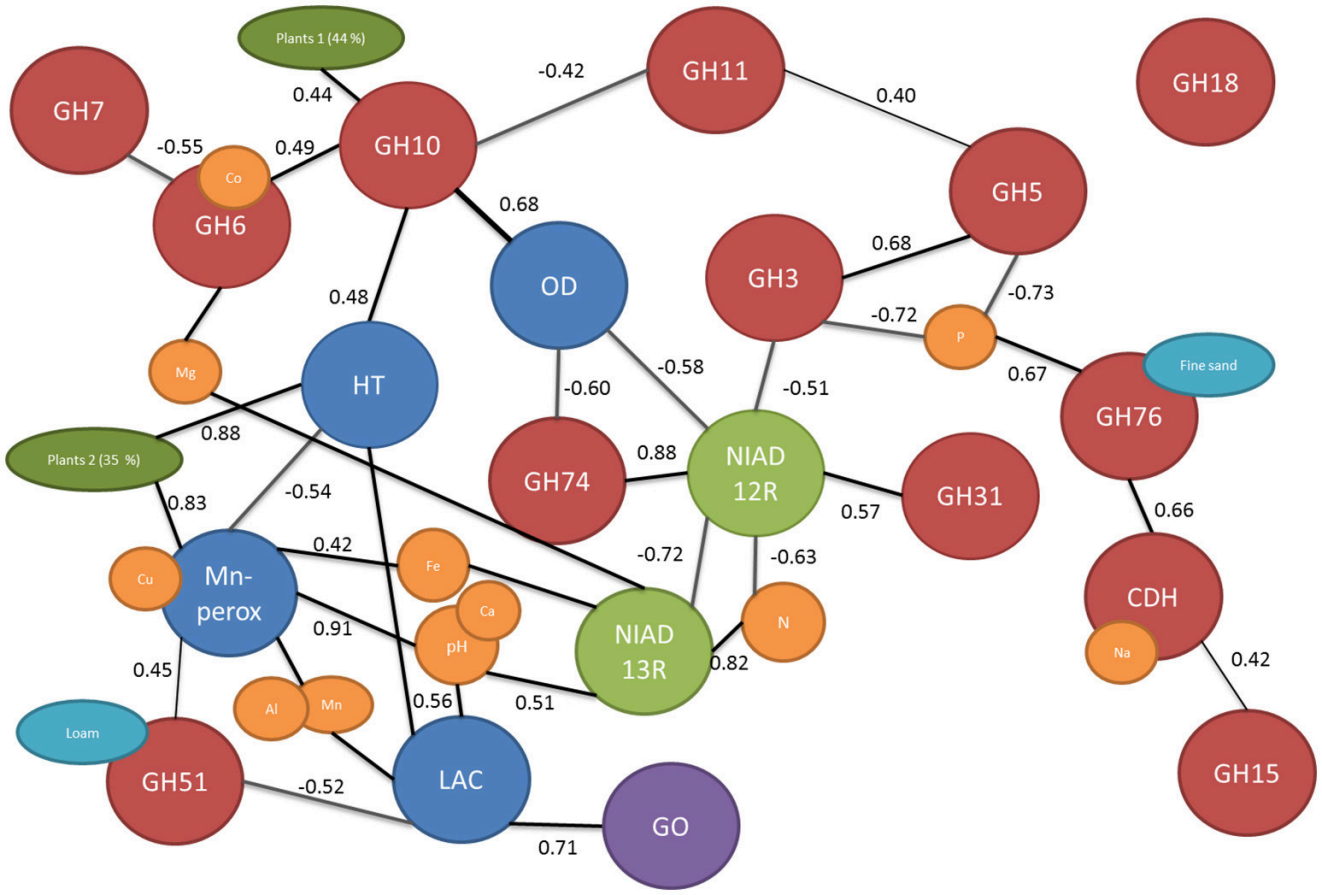
**The network of connected edaphic and biological variables based on correlation of dissimilarity matrix**. The cellulose and hemicellulose related genes are marked with red balls, nitrogen uptake genes with green, lignolytic genes with blue, glucose oxidase in purple, edaphic factors with yellow, and turquois and plant cover (calculated from PCA) in dark green. Only significant correlations are presented and the r value of the linear correlation is shown next to the connecting lines.

**Figure 3:**
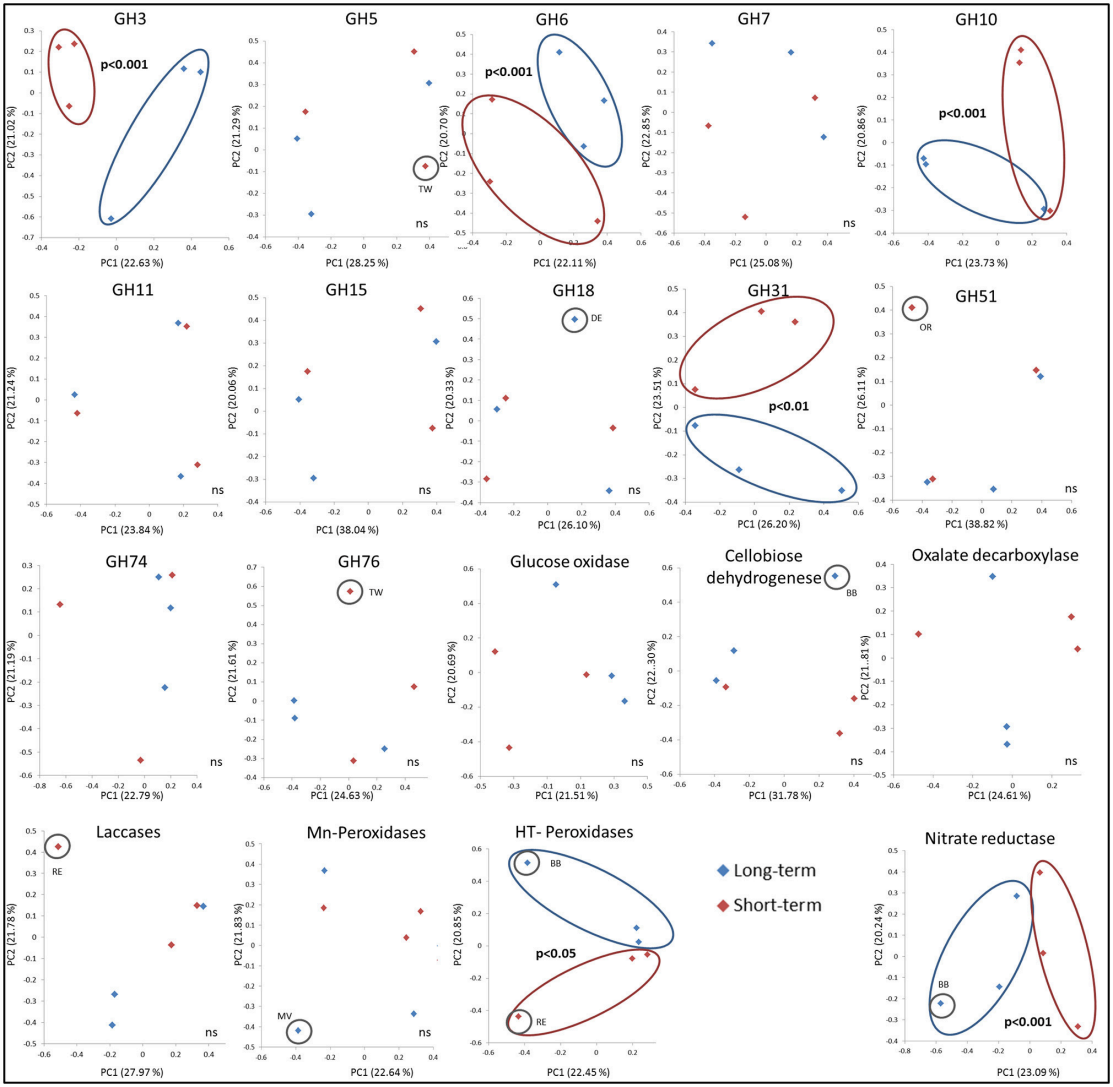
**PCA ordination of the functional community structure in long-term (blue) and short-term (red) abandoned fields based on weighted UniFrac distances**. Significance is based on UniFrac significance between treatments, is marked in the figure and significantly different treatments/samples are circled.

Due to limited numbers of sequenced clones in most of the gene families and the lack of typical grassland species in sequenced genomes, it was impossible to assign sequences from environmental samples to the genus level. In many cases, however, it was possible to assign the sequences to a certain order (with similarities of >75% to known sequenced species). The mean BLAST N (based on DNA) and BLAST P scores here varied between 55–100% and 38–95% identities, respectively. After aligning the sequences obtained in this study with selected sequences from the databases representing as diverse group of fungi as possible (Table S4), most similar reference sequence was resolved for each sequence indicating roughly to which phyla and order the type belongs to. The highest similarities with known sequences of the most common sequence type in each field are presented in Table 4. The explained differences in GH3 gene composition could be explained with the absence of sequences related to known GH3 β-glucosidases from Agaricales in short term abandoned fields where sequences related to Sordariomycetes were dominant (**Figure 3**). The separation of one field observed for GH5 could be explained by dominance of a type related to exo-β -1,3-glucanase from *Laccaria bicolor* (86–96% similarity) in all except that field. The dominant GH6 fragment type in all the long-term abandoned fields was related to a GH6 gene from Eurotiales while the most common type in short-term abandoned fields was related to type identified earlier from species belonging to Sordariales. The significant differences between short-term and long-term abandoned fields were explained by absence of few cellobiohydrolases from basidiomycete origin in short term abandoned fields. The GH10 types dominating the long-term abandoned fields were most related to basidiomycete fungus and in short-term abandoned fields dominant types were related to ascomycete xylanase explaining observed differences between the treatments (**Figure 3**). The GH31 types responsible for significant differences observed between short-term and long-term abandoned fields (**Figure 3**) were attributed to ascomycetes *Myceliophthora thermophile* and *Pyrenophora tritici-repentis*, and basidiomycete *Agaricus bisporus*, all present only in long-term abandoned fields. The GH51 types explaining the pattern seen in Figure 2 were types related to *Magnaporthales* sp. and to *Penicillium* sp. present only in short-term abandoned field OR. The dominant GH51 types in long-term abandoned fields were related to gene for α-L-arabinofuranosidase from *Pleurotus* sp. and in short-term abandoned fields α -L-arabinofuranosidase from Trichoderma.

**Table 4:** **The dominant species (and similarity %) in the fields**.

	**Long-term abandoned**			**Short-term abandoned**		
	**BB (29 years)**	**DE (29 years)**	**MV (26 years)**	**TW (9 years)**	**RE (6 years)**	**OR (6 years)**
GH3	Laccaria bicolor (92%) (KP748533)	Botryotinia fuckeliana (86%) (KP748534)	Trichoderma reesei (91%) (KP748536)	Trichoderma reesei (90%) (KP748536)	Schizophyllum commune (89%) (KP748542)	Trichoderma reesei (87%) (KP748540)
GH5	Laccaria bicolor (86%) (KP748545)‘	Laccaria bicolor (90%) (KP748548)	Laccaria bicolor (93%) (KP748550)	Trichoderma harzianum (93%) (KP748561)	Laccaria bicolor (96%) (KP748559)	Laccaria bicolor (90%)(KP748555)
GH6	Talaromyces leycettanus (84%) (KP748566)	Talaromyces leycettanus (72%) (KP748569)	Talaromyces leycettanus (78%) (KP748605)	Melanocarpus albomyces (86%) (KP748601)	Talaromyces leycettanus (83%) (KP748592)	Melanocarpus albomyces (78%) (KP748573)
GH7	Trichoderma saturnisporum (89%) (KP748613)	Trichoderma saturnisporum (89%) (KP748645)	Botryotinia fuckeliana (75%) (KP748622)	Botryotinia fuckeliana (92%) (KP748630)	Botryotinia fuckeliana (88%) (KP748636)	Botryotinia fuckeliana (87%) (KP748634)
GH10	Postia placenta (85%) (KP748716)	Postia placenta (90%) (KP748720)	Postia placenta (90%) (KP748728)	Fusarium oxysporum (78%) (KP748706)	Fusarium oxysporum (84%) (KP748704)	Fusarium oxysporum (83%) (KP748710)
GH11	Magnaporthe oryzae (87%) (KP748756)	Magnaporthe oryzae (83%) (KP748757)	Magnaporthe oryzae (91%) (KP748731)	Magnaporthe oryzae (84%) (KP748753)	Magnaporthe oryzae (89%) (KP748776)	Magnaporthe oryzae (90%) (KP748749)
GH15	Aspergillus niger (85%) (KP748779)	Cryptococcus neoformans (91%) (KP748782)	Cryptococcus neoformans (89%) (KP748782)	Laccaria bicolor (91%) (KP748784)	Fomitopsis palustris (KP748781)	Schizophyllum commune (89%) (KP748780)
GH18	Cryptococcus gattii (91%) (KP748786)	Cryptococcus gattii (91%) (KP748786)	Cryptococcus gattii (94%) (KP748782)	Cryptococcus gattii (92%) (KP748781)	Cryptococcus gattii (87%) (KP748784)	Cryptococcus gattii (84%) (KP748792)
GH31	Thielavia terrestris (92%) (KP748811)	Thielavia terrestris (86%) (KP748811)	Thielavia terrestris (89%) (KP748811)	Thielavia terrestris (89%) (KP748811)	Thielavia terrestris (90%) (KP748811)	Thielavia terrestris (89%) (KP748811)
GH51	Pleurotus sp. ‘Florida’ (87%) (KP748812)	Pleurotus sp. ‘Florida’ (83%) (KP748812)	Pleurotus sp. ‘Florida’ (89%) (KP748812)	Magnaporthe oryzae (91%) (KP748814)	Trichoderma reesei (91%) (KP748813)	Trichoderma reesei (91%) (KP748813)
GH74	Sordaria macrospora (90%) (KP748814)	Myceliophthora thermophila (91%) (KP748818)	Agaricus bisporus (89%) (KP748819)	Trichoderma reesei (90%) (KP748817)	Trichoderma reesei (93%) (KP748817)	Aspergillus niger (94%) (KP748822)
GH76	Nectria haematococca (81%) (KP748824)	Nectria haematococca (87%) (KP748837)	Aspergillus oryzae (74%) (KP748823)	Nectria haematococca (86%) (KP748831)	Aspergillus oryzae (85%) (KP748830)	Nectria haematococca (88%) (KP748827)
Glucose Oxidase	Botryotinia fuckeliana (83%) (KP748840)	Aspergillus niger (82%) (KP748855)	Botryotinia fuckeliana (88%) (KP748844)	Botryotinia fuckeliana (92%) (KP748857)	Botryotinia fuckeliana (89%) (KP748856)	Botryotinia fuckeliana (88%) (KP748838)
Cellobiose dehydrogenase	Magnaporthe oryzae (80%) (KP748663)	Magnaporthe oryzae (79%) (KP748669)	Magnaporthe oryzae (82%) (KP748663)	Magnaporthe oryzae (78%) (KP748658)	Magnaporthe oryzae (85%) (KP748667)	Magnaporthe oryzae (80%) (KP748660)
Oxalate decarboxylase	Magnaporthe oryzae (88%) (KP749156)	Botryotinia fuckeliana (82%) (KP749163)	Magnaporthe oryzae (88%) (KP749156)	Botryotinia fuckeliana (88%) (KP749163)	Botryotinia fuckeliana (86%) (KP749163)	Magnaporthe oryzae (84%) (KP749162)
Laccases	Pycnoporus cinnabarinus (88%) (KP748922)	Pycnoporus cinnabarinus (87%) (KP748922)	Rhizoctonia solani (89%) (KP748932)	Rhizoctonia solani (88%) (KP748932)	Pycnoporus cinnabarinus (82%) (KP748944)	Pycnoporus cinnabarinus (91%) (KP748922)
Mn-Peroxidase	Phlebia radiata (92%) (KP748987)	Phlebia radiata (94%) (KP749001)	Uncultured Cortinarius (97%) (KP748966)	Phlebia radiata (93%) (KP748992)	Phlebia radiata (89%) (KP749012)	Phlebia radiata (92%) (KP748977)
Hemethioperoxidase	Mycosphaerella graminicola (87%) (KP748876)	Mycosphaerella graminicola (81%) (KP748914)	Coprinopsis cinerea (87%) (KP748906)	Mycosphaerella graminicola (75%) (KP748900)	Mycosphaerella graminicola (78%) (KP748870)	Mycosphaerella graminicola (89%) (KP748895)
NIAD 12R	Fusarium oxysporum (84%) (KP749132)	Fusarium oxysporum (78%) (KP749091)	Fusarium oxysporum (77%) (KP749132)	Fusarium oxysporum (92%) (KP749106)	Fusarium oxysporum (74%) (KP749095)	Fusarium oxysporum (93%) (KP749117)
NIAD 13R	Phanerochaete chrysosporium (77%) (KP749067)	Phanerochaete chrysosporium (91%) (KP749058)	Phanerochaete chrysosporium (85%) (KP749075)	Fusarium oxysporum (81%) (KP749047)	Phanerochaete chrysosporium (78%) (KP749067)	Fusarium oxysporum (77%) (KP749074)

The representative sequence we deposited to the database is indicated in brackets.

The dominant types of laccases were from basidiomycete origin but also fragments similar to laccases from ascomycete species were detected. Field RE was significantly different from other fields mostly due to absence of *Neurospora, Aspergillus*, and *Rhizoctonia* type fragments which led to dominance of type similar to basidiomycetes (Polyporales). Mn-peroxidases in all fields were dominated with fragments from basidiomycete origin. Field MV separated due to dominance of a type similar to uncultured *Cortinarius* clone from a previous study and presence of a type categorized to Russulales (83% similarity). The dominant types of heme-thiolate peroxidases were categorized as Capnodiales in most of the fields. Both fields BB and RE had many unique types that were similar to sequences from a previous study but not closely related to known sequences from any organism (Kellner et al., 2010). The dominant types of nitrate reductases were similar to nitrate reductases from *P. chrysosporium* and *F. oxysporum* and the dominant types were not significantly different between fields. The observed differences in community structure could be explained with presence and absence of rarer gene types such as uncultured Pezizomycotina nitrate reductase (HQ243641) that was primary present in fields abandoned for short time.

There were significant differences in functional gene composition between the fields which could not be explained by time since agriculture (Figure 3). Thus, the dissimilarity of the fields to each other were calculated using UniFrac distances and correlated with dissimilarities between measured environmental parameters. Significantly correlated dissimilarities are connected with lines or overlapping balls between genes and environmental parameters in the network Figure 2. Furthermore, to learn about connections between the genes, the dissimilarities were correlated with each other and significant correlations were marked with lines connecting the genes (Figure 2). There were total of 19 significant dissimilarity correlations between gene fragments (Figure 2). Eight of these correlations were negative and 11 positive. All other genes except GH18 family chitinases were correlated with at least one other gene or environmental factor. Cellobiohydrolase II gene (GH6) fragment dissimilarity was negatively correlated with cellobiohydrolase I (GH7) fragment dissimilarity. Endo-β-1,4-xylanase dissimilarities of families GH10 and GH11 were also negatively correlated with each other.

Mn-peroxidase and laccase fragment composition dissimilarity between fields was positively correlated with the dissimilarity in pH and associated (metal) ion contents in soils (Figure 2). pH differences in soils further affected the basidiomycete community responsible for nitrate uptake. Nitrogen content dissimilarity in soils (calculated dissimilarities between soils in their nitrogen content) was correlated with dissimilarity of the fungal genes responsible for nitrate uptake. The nitrate reductases from basidiomycetes were positively correlated with dissimilarity of nitrogen contents in soils while ascomycete nitrate reductases were negatively correlated. GH5 and GH3 were negatively and GH76 positively associated with soil phosphorous dissimilarity in soils (Figure 2). Dissimilarity in plant cover between fields calculated from principal component (PC) axis explaining 79% of the variation in plant community (44 and 35% for PCA1 and PCA2, respectively). The plant cover expressed using the PCA axis was related to GH10, Heme-thiolate peroxidase, and Mn-peroxidase composition dissimilarities in the soils.

## Discussion

In contrast to the commonly used analyses of soil fungi using phylogenetic markers such as ITS genes (Lindahl et al., 2013), we investigated the presence and composition of 20 functional genes encoding mostly carbon cycling related and one nitrogen related genes. To achieve a goal to evaluate functional shifts in soil fungi along a chronosequence of soils abandoned from agriculture, new primers were developed and existing primers tested. An average of 138 unique sequences derived from 19 functional genes was obtained per soil sample which makes it higher coverage than obtained in metatransctiptomics or metagenomics studies. For example in a study by Damon et al. (2012) maximum of six types of genes from any given GH family were found in a sample. The genes studied here were all pre-selected for their presence and importance in soils and are involved in crucial soil processes such as cellulose, hemicellulose, and lignin degradation and nitrate reduction in which fungi are key agents. By selecting a broader range of genes as targets, we could inspect the community richness and function as a whole instead of focusing on only one or few genes of interest (Edwards et al., 2008; Kellner et al., 2009).

Unfortunately, the majority of the types of genes detected in this study could not be linked to a known species. This is an expected result as public databases have limited number, often <50, of correctly annotated reference genes and further reinforces the idea that identities of most of the genes from soil fungi are yet unknown: sequences produced in this study with highest similarities to known sequences were GH7 fragments and this was only due to the large amount of environmental sequences of cellobiohydrolases in databases. When using an approach of aligning gene fragments (mRNA) from this study to annotated genes (mRNA) from sequenced species, the similarities were in average around 85%. These results are in the range of other studies finding that average BLAST P scores obtained were around 80% for GH7 cellobiohydrolase (e.g., Edwards et al., 2008). Here, using cloning and subsequent Sanger-sequencing it was not needed to select for certain sized fragments allowing us to capture gene fragments from as diverse origin as possible. This is an advantage of this technique compared to for example some next generation sequencing (NGS) techniques such as Illumina HiSeq or MiSeq, and pyrosequencing. These techniques will generate more sequences but only fairly short and uniformly sized amplicons can be sequenced (Goodwin et al., 2016). Here, we detected large variation in intron positions and resulting amplicon sizes making the techniques sensitive to this variation less suitable for this purpose.

In some cases, the primer sets are selective to certain group of organisms. Furthermore, there is a need for primer sets that amplify fungi but exclude bacterial and plant derived sequences. Some of the primers used in this study are known to preferentially amplify only ascomycetes (GH3 and GH76) or basidiomycetes (i.e., Mn-peroxidases; Kellner et al., 2010) and our data concurs with these view (Table S3). This is primarily due to the natural lack of conservation between orders and phyla and technical design issues such as avoidance of degeneracy and limitations in the amount of reference sequences (Kellner et al., 2010). Yet, we feel that the primer sets designed in this study will enable further characterization of unknown fungal genes in soils and the long sequences generated here enable further primer development for the further capture of larger part of the soil functional biodiversity.

The depth of analyses for individual genes was rather low in this study especially when considering the diversity of soil fungi detected using analysis of ITS region (Thomson et al., 2015) and it is likely that some more quiescent groups of fungi might have not been detected. Nevertheless, the strength of this study is the multigene approach proposed and we are confident that we have captured the dominant fungal functional genes in these soils and increased, in some cases even doubled, the amount of sequences derived from soil and deposited in databases in the case of all the genes. Using this integrated multi-gene approach, we showed that the total number of fragments identified was affected by the time since abandonment from agriculture (Figure 1). The long-term abandoned fields had higher total number of unique fragments when all genes involved in cellulose, hemicellulose, and lignin degradation studied were calculated together. This could indicate that these soils abandoned for over 25 years have a larger pool of potential functions and that this functional diversity would increase in time since agriculture. Earlier study using ITS-region as marker found no significant differences between recent and long-term abandoned fields in their alpha-diversity of fungi (Thomson et al., 2015). This is in accordance with results presented here when single marker genes were used (Table 2) but contrary to the results obtained using a multi-gene approach (Figure 1).

By using ITS region as a marker, difference in community structure between short- and long-term abandoned fields was detected (Thomson et al., 2015). Here we could show that the community composition of GH3, GH6, GH10, GH31, heme-thiolate peroxidase, and nitrate reductase genes was different between short- and long-term abandoned fields making them good candidates for functional screening of soils showing differences corresponding to ITS-region. The reason why we detected changes in these genes but not in the community structure of GH5 and GH11 for example, is unclear. One possible explanation is that different fungal species possess different sets of CaZymes (Veneault-Fourrey et al., 2014) and that the diversity of the community producing certain enzymes is very small (i.e., only basidiomycetes), making it likely that differences in the community structure do not exist or are not large enough to be detected. For example, laccases can be produced by asco- and basidiomycetes and some bacteria (i.e., actinomycetes) while Mn-peroxidases are produced only by basidiomycetal fungi (Baldrian, 2006; Bödeker et al., 2009). Surprisingly, in our soils the cellobiose dehydrogenases were mostly ascomycetal types and involved in cellulose decomposition even though earlier studies claiming them to be predominantly basidiomycetal and involved also in lignin degradation (Harreither et al., 2011). This finding is related to the use of new primers which target broader range of fungi and especially ascomycetes and highlights the importance of development of primers to target broader groups of fungi in order to gain better understanding of the function of the soil fungal communities.

For GH3 and GH10, we saw a clear shift in communities in phylum level: in short-term abandoned soils the dominant types of this gene were related to ascomycetes while long-term abandoned fields were dominated with basidiomycetal types. In case of GH6 the corresponding shift explaining the observed differences between treatments was within Ascomycota (i.e., from Eurotiales to Sordariales). These are major shifts and partly corroborated with the changes observed in the taxonomic study of the same soils (Thomson et al., 2015). Probably these are a relic of the supply of substrates, but as this is not measured here, we can only speculate. Earlier studies have found that GH7 composition or richness in soil or litter is not significantly affected by tree species (Edwards et al., 2008), genetic modification of potato leaves (Hannula et al., 2013), elevated CO_2_ (Hassett et al., 2009), or N fertilization (Weber et al., 2012). Our results corroborate the lack of differentiation power of the GH7 gene to environmental disturbances. Based on evidence gathered here, we recommend that instead of focusing on one gene, studies should include multiple genes in the analyses to determine which ones are the most responsive to the disturbance in question.

The difference observed in heme-thiolate peroxidase composition can be explained by the observed differences in plant cover: fresh plant litter is rich in phenolics with high solubility and this varies greatly between plant species (Meier et al., 2008) and heme-thiolate peroxidases are involved in breakdown of these aromatic compounds while the other lignolytic enzymes are used to degrade lignin. Here we observed no differences in the composition of fungal genes involved in lignin degradation between the short-term and long-term abandoned fields. Earlier studies have found large differences in the presence and activity of genes involved in lignin decomposition between sampling times (Kellner et al., 2009), along soil depth gradients (Snajdr et al., 2008a) and between sampling sites (Snajdr et al., 2008b) in forest soils. The difference between our study sites and the earlier studies is that we were investigating grassland soils where the importance of lignocellulolytic genes might be overshadowed by the cellulolytic activities and the so-called r-strategist fungi.

By adopting an approach to look at the dissimilarity correlations between the genes and environmental factors, we could tease out the connections between the genes studied and edaphic factors (Figure 2). It is known that soil characteristics affect laccase gene composition (Lauber et al., 2009; Chen et al., 2013) and especially pH is known to influence the activity of ligninolytic enzymes in soils (Fujii et al., 2013). Furthermore, in pure culture studies it has been shown that ligninolytic enzymes have different ranges of optimum pH (Wong, 2009). We acknowledge that the use of DNA based methods tells only about the presence of these genes and not about actual production/activity of the enzyme but we are confident based on earlier research that these factors correspond well (Chen et al., 2013). Here, we detected a strong dependence of both laccases and Mn-peroxidases on the pH of the soils (Figure 2) and the conclusion may, thus, be drawn that the soil pH is the most important factor governing the composition of ligninolytic enzymes in these soils. The composition of GH3, GH5, and GH76 genes seemed to respond to differences in soil phosphorous content while, for example, sodium dissimilarities were correlated with cellobiose dehydrogenases. Logically, the nitrogen uptake genes were related to soil nitrogen content and could thus be used to study the effects of e.g., nitrogen fertilization on soil communities (Gorfer et al., 2011). Another factor affecting basidiomycetal nitrogen is, again, soil pH (Figure 2). Indeed, we can conclude that processes dominated by basidiomycetes were strongly affected by the soil pH while other soil and plant related factors were shaping the predominantly ascomycetal processes. Interestingly, nitrogen uptake was one the genes/process affected by time since abandonment and was tightly linked with dissimilarities in nitrogen contents in the soils. No gradient of nitrogen content was present in the soils but it has been hypothesized that the N mineralization is more important in later successional stages (Holtkamp et al., 2011) which coincides with increased importance of fungal communities in this system.

Fungi use a multitude of enzymes in synergy to break down plant cell walls and thus connections among genes are to be expected. For example, oxidoreductive enzymes such as cellobiose dehydrogenase can act synergistically with canonical GHs, accelerating the breakdown of litter (Langston et al., 2011). On the other hand, we saw that also genes producing similar enzymes (such as cellobiohydrolases GH6 and GH7) group together (Figure 2). The gene family GH18 involved in production of fungal chitinases was the only family not related to any edaphic factor or other gene. This is in accordance with the assumption that this gene may be involved in degradation of fungal hyphae and not involved in the decomposition of plant derived material (Hartl et al., 2012). Surprisingly, oxalate decarboxylase composition was not related to lignin degradation pathways even though it is known to be involved in regulation of intra- and extracellular quantities of oxalic acid, which is one of the key components in biological decomposition lignin (Mäkelä et al., 2009). In this system the composition of oxalate decarboxylases seems to, however, be linked to GHs from families 10 and 74 and to nitrogen uptake by Ascomycota. The cellobiose dehydrogenenase was also grouping with cellulose degradation process instead of lignin decomposition despite its known involvement in both processes (Hyde and Wood, 1997).

Using this targeted approach, we obtained over 100 unique sequences involved in carbon and nitrogen cycling per soil type which we used to characterize both the richness and community composition of the soils. The main finding is that different processes in soils are driven by different edaphic factors (such as pH) and that conversion of former agricultural land into species rich grassland affects (via changes in plant cover) the cellulolytic fungal community rather than the community that is involved in the decomposition of recalcitrant materials such as lignin.

## Author contributions

SEH and JAvV designed the experiments and wrote the manuscript. SEH performed the experiments and did the statistical analysis.

### Conflict of interest statement

The authors declare that the research was conducted in the absence of any commercial or financial relationships that could be construed as a potential conflict of interest.
