# Supplementary material for: Primer Sets Developed for Functional Genes Reveal Shifts in Functionality of Fungal Community in Soils
Source: Front Microbiol. 2016 Nov 29;7:1897. doi: 10.3389/fmicb.2016.01897 (PMC5126076; doi:10.3389/fmicb.2016.01897)
Supplement: Supplementary file 2 [file Table2.docx]

Table S1: Field properties, location and time since abandonment

Table S2: The primers and PCR conditions used.

*denotes the primer finally used when multiple options were tested

Table S3: The fungal strains used to test primers giving positive and negative signals for PCR and their identity (% similarity) based on ITS sequencing

Table S4: The reference strains to which sequences obtained in this study were compared with.
